# Subcritical Extracts from Major Species of Oil-Bearing Roses—A Comparative Chemical Profiling

**DOI:** 10.3390/molecules26164991

**Published:** 2021-08-18

**Authors:** Ana Dobreva, Daniela Nedeltcheva-Antonova, Nenko Nenov, Kamelia Getchovska, Liudmil Antonov

**Affiliations:** 1 Institute of Roses and Aromatic Plants, Agricultural Academy, 6100 Kazanlak, Bulgaria; anadobreva@abv.bg; 2 Institute of Organic Chemistry with Centre of Phytochemistry, Bulgarian Academy of Sciences, 1113 Sofia, Bulgaria; k_candeva@abv.bg; 3 Department of Heat Engineering, Technical Faculty, University of Food Technologies, 4002 Plovdiv, Bulgaria; nenonenov@e-xtracts.com; 4 Institute of Electronics, Bulgarian Academy of Sciences, 1784 Sofia, Bulgaria; liudmil.antonov@gmail.com

**Keywords:** subcritical freon extraction, GC/MS, aromatic plants, oil bearing roses, 2-phenyl ethyl alcohol, terpenes

## Abstract

A comprehensive chemical profiling of 1,1,1,2-tetrafluoroethane (freon R134a) subcritical extracts from the main genotypes of oil-bearing roses, was performed by gas chromatography–mass spectrometry (GC/MS) and gas chromatography with flame ionization detection (GC-FID) in order to reveal the differences in their chemical composition. One hundred and three individual compounds were identified using GC/MS and their quantitative content was determined using GC-FID, representing 89.8, 92.5, 89.7 and 93.7% of the total content of *Rosa gallica* L., *Rosa damascena* Mill., *Rosa alba* L. and *Rosa centifolia* L. extracts, respectively. The compounds found in the extracts are representatives of the following main chemical classes: mono-, sesqui- and triterpenoids, phenylethanoids and phenylpropanoids and aliphatic hydrocarbons. Fatty acids, esters and waxes were found, as well. The study revealed that 2-phenylethanol is the most abundant component, ranging 9.0–60.9% followed by nonadecane and nonadecene with 5.1–18.0% geraniol (2.9–14.4%), heneicosane (3.1–11.8%), tricosane (0.1–8.6%), nerol (1.3–6.1%) and citronellol (1.7–5.3%). The extracts demonstrate a specific chemical profile, depending on the botanical species—phenylethanoids and phenyl propanoids are the main group for *R. damascena*, aliphatic hydrocarbons for *R. alba* and *R. centifolia*, while both are found in almost equal amounts in *R. gallica*. The terpenoid compounds show relatively broad variations: monoterpenes—11.9–25.5% with maximum in *R. centifolia*; sesquiterpenes—0.6–7.0% with maximum in *R. gallica* and triterpenes—0.4–3.7% with maximum in *R. gallica* extract.

## 1. Introduction

The essential oils and extracts of oil-bearing roses are high-value natural products with a broad range of applications. They are indispensable in fine perfumery and cosmetics, as food additives and aromatherapy agents [1]. Nowadays, several species, namely, *R. damascena* Mill., *R. gallica* L., *R. centifolia* L. and *R. alba* L., are used for the commercial manufacturing of rose oil, rose water, absolute and concrete [2,3]. Rose aroma products are well known for their perfumery properties and rich pharmacological activity, showing antioxidant, antimicrobial, analgesic, antispasmodic, muscle relaxant, anti-inflammatory, anticonvulsant and antiviral effects, as described in the literature [2,3,4,5,6,7,8]. Bulgaria is the only country, where the industrial cultivation of *R. alba* and *R*. *damascena* takes place, along with small areas of *R. gallica* and *R. centifolia* [4,9]. The quality of the rose oil is standardized according to ISO9842 [10]. Turkey, Iran, India, Afghanistan, China and Saudi Arabia grow *R. damascena*, while Morocco cultivates mainly *R. centifolia*. In the northern countries, where the frost resistance plants are preferred (Russia, Ukraine, Moldova), *R. gallica* is mainly spread. Each of the rose genotypes has its own advantages, which are used in breeding and selection programs, resulting in crossbreeds (subsequently varieties) aimed to bring high economic value.

The aroma composition of rose oil has been investigated intensively [11,12,13,14,15,16,17,18,19,20,21,22], but alternatively, the rose scent could be extracted as a wax-like substance called rose concrete by non-polar solvent extraction (usually -n-hexane), which, followed by ethanol re-extraction, produces rose absolute [11,23]. Following the production process, it is considered that the rose absolute much better reflects the chemical composition of the rose flower [24,25]. There are, currently, few investigations of the rose absolute chemical composition, which are focused only on a few main constituents and predominantly on absolutes produced in Turkey [6,15,23,26,27,28,29].

The modern green-economy puts additional requirements for the solvents used in the extracts production, leading to a general trend to reduce or eliminate petrochemical solvents and to decrease energy consumption in addition. Therefore, an “eco-footprint” was determined for each solvent, based on the six principles of green extraction defined by Chemat and co-workers [30]. The application of liquefied gases brings additional advantages over traditional extraction techniques such as the flexibility of the processing and the lack of the residual solvent contamination of the final product, which eliminates the expensive post-processing treatment.

Since the last decade, 1,1,1,2-tetrafluoroethane has been widely used as a solvent for the extraction of valuable natural compounds from aromatic and medicinal plants [31,32,33,34,35]. The products of this type of extraction could be used as food grade aroma preparations according to existing regulations [36]. Except some environmental and cost consideration, 1,1,1,2-tetrafluoroethane is close to an ideal solvent for extraction due to the following:

- High selectivity to smelling natural compounds (i.e., terpenes and their derivatives) [31,34,35];
- Very high chemical resistance—no interaction with the processed plant, its compounds and equipment walls (high quality and purity of final product) [37];
- Low viscosity and surface tension of liquid solvent [37]—intensive extraction process for short extraction times, even at low temperatures;
- Low extraction temperatures [31,34,35]—close to ambient ones (no thermal degradation of heat sensitive compounds during extraction and separation and hence high aroma quality of final product);
- Low boiling points [37]—easy and effective separation of final product from solvent;
- Low values of vaporization heat [37]—high level of energy efficiency and low energy input for micellar separation;
- Absence of own smells and taste [38]—high purity of final extracts;
- Safe for the human health [38] and suitable for food-grade aroma preparations;
- Fire and explosion safe properties [38];
- Moderate extraction pressures [31,34,35]—relative light, low cost extraction equipment.

The hot compressed water is also used as an alternative for the extraction of valuable natural compounds. The use of hot compressed water can be advantageously exploited over a wide range of temperatures and appropriate pressures to cover extraction with targeted substrates. It is a new and powerful technique at temperatures between 100 and 374 °C and a pressure high enough to maintain the liquid state [39]. Its high boiling point for its mass and high dielectric constant make it most efficient to high polar target materials, whereas moderately polar and non-polar targets (as fragrances) require a less polar medium induced by an elevated temperature. The subcritical extraction of dried *R. damascena* flowers resulted in a high yield (38.14%), but the quality of the product is unsatisfactory [40]. Solvent-free microwave-assisted extraction and ohmic-assisted hydrodistillation are advanced and green distillation techniques, recently reported as promising alternatives in the extraction of rose aroma products [41].

Comparing the solvents for rose absolute production, the supercritical CO_2_ extraction of rose concrete was found to be perspective, giving a yield of 2-phenyl ethanol over 50% larger [42] in respect of traditionally used ethanol [43,44]. The extraction of *R. damascena* with CO_2_ (25.5 MPa) and co-solvent ethanol was used alternatively to produce quercetin [45]. Wilde and McClory [46] described the application of 1,1,1,2-tetrafluoroethane (known also as freon R134a) for the subcritical extraction of *R. damascena*, but details about the extract were not provided.

Baser et al. [47] reported extraction with a mixture of 90% tetrafluoroethane:10% diethyl ether, mentioning the importance of the extraction time. They found that 2-phenylethyl alcohol was the main component in almost all the extraction periods (45 min, 5, 8 and 24 h) with the best yield (69.6%) achieved at 45 min. The total terpenoids content decreases during prolonged extraction, while that of the paraffin’s increases, as expected. A comparative study of rose oil obtained through traditional technology and extracts obtained using supercritical CO_2_ and R134a shows that the chemical composition of the extracts was dominated by 2-phenylethyl alcohol (56.6% in the subcritical sample and 46.7% in the supercritical one), while the main aroma constituents were found substantially higher in the oil [48,49]. A comparison between 1,1,1,2-tetrafluoroethane and the more polar CO_2_ indicates that the former allows work at a lower pressure leading to much less degradation of the final products [50].

The chemical composition of the rose extracts, similarly to the rose essential oil, should be strongly affected by the botanical and geographical origin of the raw plant material, environmental conditions, the production method (the extraction selectivity of the liquefied gases, for example) and storage. However, up to now, these effects have not been systematically studied. Therefore, the aim of the current study is to investigate and compare the chemical profile of R134a extracts from the main commercially grown oil-bearing rose species, namely, *R. damascena* Mill., *R. gallica* L., *R. centifolia* L. and *R. alba* L., by means of gas chromatography. To the best of our knowledge, no such systematic comparative study has been performed up to now. The acquired quantitative data could have substantial, fundamental and practical value.

## 2. Results and Discussion

**Figure 1:**
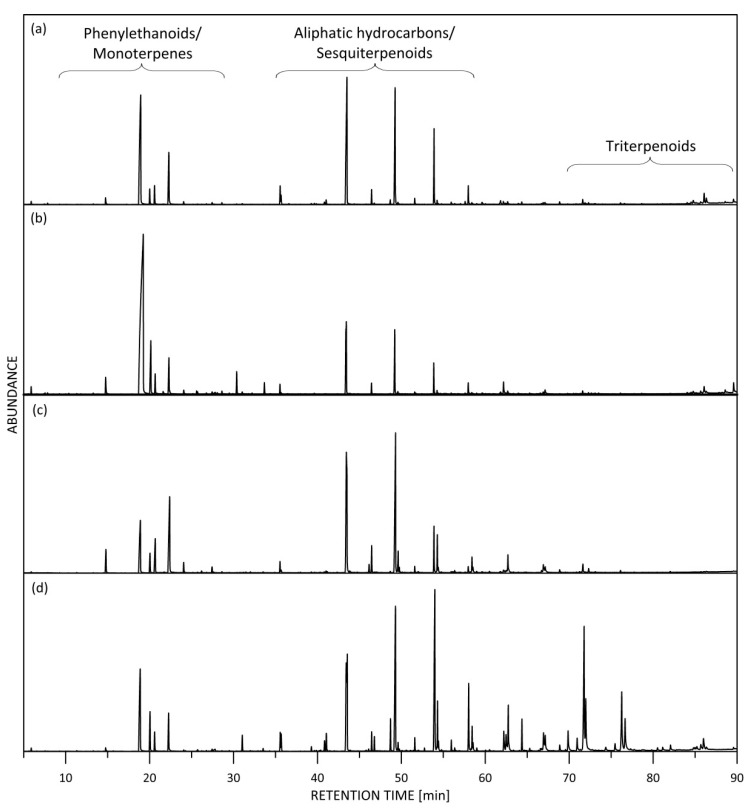
GC/MS profile of subcritical (1,1,1,2-tetrafluorethane) extracts on a DB-17HT column: (**a**) *R. gallica*; (**b**) *R. damascena*; (**c**) *R. alba*; (**d**) *R. centifolia*.

As a result of the analysis, more than 150 compounds with concentrations higher than 0.01% were detected in the rose subcritical extracts and 103 of them, containing C7-C30 carbon atoms, were identified using GC/MS and simultaneously quantified using GC-FID. In Figure 1, the Total Ion Current (TIC) chromatograms of the R134a extracts are compared. The quantitative content, as determined using GC-FID, is shown in Table 1.

As seen from Table 1, the main constituents or rose extracts are representatives of the phenylethanoids and phenylpropanoids, aliphatic hydrocarbons and terpene compounds (mono-, sesqui- and triterpenoids). Fatty acids, higher alcohols and waxes were found as well.

It is worth mentioning that the studied extracts show a similar qualitative content with different quantitative characteristics. In general, according to our current and previous [24] results, the chemical profile of the subcritical extracts is closer to the rose absolute, than to the rose oil.

### 2.1. Phenylethanoids and Phenylpropanoids

2-Phenylethanol is one of the abundantly emitted scent compounds in rose flowers, responsible for the characteristic odour of rose. In the frame of the current study, 2-phenethyl alcohol is found to be the main component in the subcritical extracts, ranging from 9.0 (*R. centifolia*) to 59.6% (*R. damascena*). This content is comparable with the average concentration of 46.56 ± 0.18% in the *R. damascena* absolute, while it is only 0.66 ± 0.01% in the rose oil [24]. The results could be explained by the specificity of 2-phenethyl alcohol biosynthesis in each rose genotype [51,52], but also by environmental factors and storage conditions.

Phenylpropanoids (eugenol and methyl eugenol) are naturally occurring compounds in a number of oil-bearing species and essential oils, including roses and rose oil, with a definitely positive scent contribution.

Eugenol was found in *R. damascena* in the amount of 1.3% and as a trace component (<0.1%) in *R. gallica and R. alba extracts*.

In the last few decades, concerns were raised by the European Commission’s Scientific Committee on Consumer Products about the potentially allergic and carcinogenic effects of methyl eugenol [53]. Therefore, substantial efforts have been made to reduce its content in cosmetics and especially in food. In our study, methyl eugenol was detected only in the *R. damascena* extract, in the amount of 0.5%. By comparing this result with the existing data for CO_2_ extracts [43,44], it could be concluded that, in contrast with other extractants, R134a has no affinity for this compound [28] and could be used for the production of low methyl eugenol content products. The other studied rose species, except *R. centifolia*, are known to biosynthesize methyl eugenol in insignificant amounts [54], which was also reported for their essential oils [55,56] and has been confirmed for the subcritical extracts in the current study.

### 2.2. Terpenoids

Terpenes and terpenoids are the main biosynthetic building blocks and important mediators of ecological interactions in the plants. They are the primary constituents of the essential oils.

#### 2.2.1. Monoterpenes and Their Oxygenated Derivatives

*Monoterpene hydrocarbons*, with main representatives α- and β-pinene, β-myrcene and limonene, are detected in low concentrations in all the samples from 0.2% in *R. centifolia* to 0.6% in *R. damascena* extract.

The acyclic alcohols geraniol, nerol and citronellol are the main monoterpene alcohols observed in the extracts. *R. alba* reached the maximum with amount of 14.4% for geraniol and 6.1% for nerol, while *R. damascena* shows the highest concentration of 5.3% for the citronellol. These results correspond to the literature data for rose concrete [28], CO_2_-extract and absolute [43,44]. It is worth noting that the main terpene alcohols, despite being observed in lower amounts, show the same citronellol:geraniol:nerol ratio as in the corresponding essential oils [2,55].

Another monoterpene alcohol found in the extract is linalool as a trace component (<0.1%) in all the extracts.

*Monoterpene esters.* Several geranyl and citronellyl esters (primarily as formates, acetates and propionates, but also as long chain organic acids esters) were found in a very broad range, from 0.3% in *R. gallica* to 17.7% in *R. centifolia* (Table 1).

#### 2.2.2. Sesquiterpenes

Sesquiterpene hydrocarbons are found in a relatively low concentration, with the following main representatives: β-caryophyllene, germacrene D, β-cubebene, β-elemene, β-cadinene, etc. In respect of the total sesquiterpene content, the extracts could be divided in the following two groups: the first one with a higher concentration of 7.0% for *R. gallica* and 5.2% for *R. centifolia,* and the second, with a substantially lower amount of 2.1% for *R. alba* and 0.6% for *R. damascena*.

#### 2.2.3. Triterpenoids

Triterpenes are one of the most numerous and diverse groups of plant natural products. Simple triterpenes are components of surface waxes and membranes and may potentially act as signalling molecules. Triterpenes have a broad spectrum of biological activity: antioxidant, anticancer, antibacterial, antivirus, gastroprotective, hepatoprotective, antipancreatitic, anticholytic, antihyperglycemic and hypolipidemic effects [57] and have a wide range of applications in the food, health and industrial biotechnology sectors.

The most frequently found triterpenoids in the rose species are lupeol, α-amyrin, β-amyrin, oleanolic acid, ursolic acid, carotenoids and tocopherols [58]. In our study, we identified eight triterpene compounds; among them most abundant are lupeol (1.2% in *R. damascena* and 0.7% in *R. gallica*) and β-amyrin (0.9% in *R. gallica* and 0.5% in *R. damascena*). The total amount ranges from 2.7 to 3.6%. The maximum is in *R. gallica*, in *R. damascena* and *R. centifolia* the level is equal, while in *R. alba* they are found in the amount of only 0.4%. Up to date, there were no data in the literature for identified triterpenes in CO_2_ extracts [42,43,44,59], while for the rose absolute, the values are comparable [24].

### 2.3. Aliphatic Hydrocarbons (Stearopten)

Aliphatic hydrocarbons (saturated and unsaturated) are the main building blocks on the surface of rose flowers. Aliphatic hydrocarbons, despite being odourless, play an important role in the rose extracts’ chemical composition as compounds, responsible for the odor stability, i.e., they serve as odor fixators. The content of heptadecane and nonadecene/nonadecane is considered to be of particular importance. The total content of alkanes and alkenes found in the rose extracts is in the range from 12.8% in *R. damascena* to 41.7%. in *R. centifolia*. The most abundant are nonadecane and nonadecene with 5.1 ÷ 17.9%, followed by heneicosane (3.1 ÷ 11.8%), tricosane (0.1 ÷ 8.6%), pentacosane (0.5 ÷ 2.6%) and heptadecane—0.5 ÷ 1.5%. The distribution of aliphatic hydrocarbons in the extracts of the different roses show the same pattern as in the essential oils, but in lower values [2,55].

### 2.4. Others

*Fatty acids, alcohols and aldehydes.* These compounds, naturally presenting in the rose flowers, are normally not observed in the essential oil samples due to their low volatility, but they were found in relatively high amounts in the subcritical extracts (from 0.4% in *R. centifolia* to 5.0% in *R. alba*), which could be explained with the extraction selectivity of the R134a used as an extractant.

**Figure 2:**
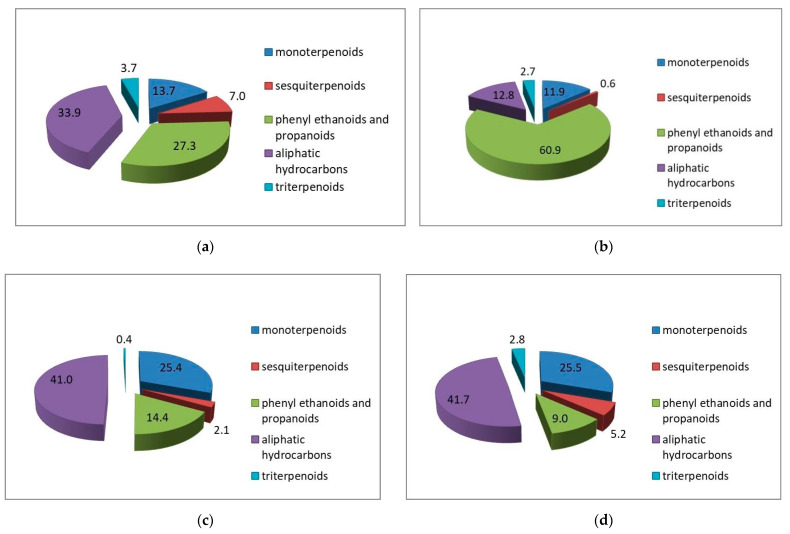
Main chemical classes distribution, according to the data from Table 1: (**a**) *R. gallica*, (**b**) *R. damascena*, (**c**) *R. alba* and (**d**) *R. centifolia*.

The components’ distribution in different chemical classes in the subcritical rose extracts is presented in Figure 2.

As seen from Figure 2, *R. alba* and *R. centifolia* extracts have very similar chemical profiles dominated by the saturated and unsaturated aliphatic hydrocarbons (41.9 and 41.7%, respectively), followed by monoterpenes (25.4 and 25.5%, respectively), phenylethanoids (14.4 and 9.0%, respectively) and sesquiterpenes (2.1 and 5.2%, respectively). *R. damascena* extract is the richest in phenylethanoids with 60.9%, followed by aliphatic hydrocarbons (12.8%) and monoterpenes (11.9%), with a minimum of sesquiterpenes content of 0.6%. The extract of *R. gallica* has almost an equal quantity of aliphatic hydrocarbons and phenylethanoids (33.9 and 27.3%, respectively), the next are monoterpenes (13.4%) and the last are sesquiterpenes (7.0%).

## 3. Materials and Methods

### 3.1. Raw Plant Material

The experiment was conducted in 2019. The plants of *R. damascena* Mill., *R. gallica* var. *officinalis* Thory., *R. centifolia* L. and *R. alba* L. (Figure S1) are part of a roses collection in the experimental field of Institute for Roses and Aromatic Plants, Kazanlak, Bulgaria. The fresh blossoms were picked up in the morning (6–10 a.m.) in the most appropriate development phase, according to Staikov et al. [60]. Due to the different flowering periods of the species, the raw material was kept by freezing at −20 °C until processing, which, according to Seify et al. [61], is the most appropriate storage method, best preserving the raw material quality. Pictures of the raw material of *R. damascena* Mill. before and after extraction as well as of the obtained products are shown in Figure S2.

The moisture content of the material was 80–82%, measured by drying to constant weight.

### 3.2. Extraction

Extraction of the raw material using 1,1,1,2-tetrafluoroethane was performed on a pilot apparatus. The unit consists of a 1-liter extraction vessel, a 5.5-liter collector vessel, equipped with a 200 W electric heater, compressor and heat exchanger unit, and a filtration set. The system is equipped with temperature and pressure sensors. It is controlled by a fully automatic Programmable Logic Controller (PLC) screen interface with first level safety functionality and user programmable parameters (extraction pressure, number of extractions, separation end pressure and extraction time). The heating mantle was constructed around a collector vessel to maximize the freon transfer rate from liquid to gas state.

The used extraction equipment, model TFE-EXV-1L (www.e-xtracts.com, accessed on 14 August 2021) is produced by InnoSolv ltd., Plovdiv, Bulgaria. Most of the details for the working principles and main part of the equipment are confidential according to signed third party agreements. From a scientific point of view, the equipment features are static raw material and the percolation of solvent by gravity with continuous removing of miscella and adding of fresh solvent, initial vacuuming of extractor and raw material, solvent recovery of residue, isobaric process (equal solvent pressure in system extractor/evaporator/condenser), etc.

Periodic stationary extractions were carried out using 300 g of plant material for a single charge. The raw material was sputtered with solvent and after double extraction at 0.5–0.6 MPa and temperature 20–25 °C, the supernatant was drained into a separator, where the solvent was evaporated at a lower pressure, and in the last few minutes, low heat was used to completely eliminate the vapor.

Extraction conditions were as follows: liquid/solid ratio of 4.0 BV/h, solvent conditions in the extraction chamber being thermodynamically close to saturated, boiling liquid at pressure 0.73–0.77 MPa and corresponding saturation temperature in range 28–30 °C, single extraction step, extraction time was 40 min. As the process is an isobaric one, the separation pressure is equal to the extraction pressure.

The extraction parameters were chosen according to our previous experience and different plants trials for the extraction of targeted smelling compounds [31,34,35] (for heat resistant, low molecular weight compounds usually pressure in range 0.6–1.0 MPa and extraction time between 30 to 60 min).

A food grade 1,1,1,2-tetrafluoroethane (CAS number 811-97-2), purchased from the Frigo Chem Ltd. (Bulgaria), was used as an extractant. It is non-polar, pressurized gas solvent featuring colourless and practically odourless liquid with the following parameters: dipole moment of 2.058 Debay [38]; dielectric constant of 3.54 [62]; boiling temperature of −26 °C at 0.101 MPa; saturation pressure at 20 °C of 0.57 MPa [37]; dynamic viscosity at 20 °C of 0.2 mPa; surface tension at same temperature 8.5 mN/m [37].

### 3.3. Analysis

#### 3.3.1. Gas Chromatography-Mass Spectrometry (GC/MS)

The GC/MS analysis was performed on an Agilent 7820A GC System Plus gas chromatograph coupled with 5977B Mass Selective detector and flame-ionization detector (Agilent Technologies, Palo Alto, CA, USA). A fused silica capillary column, a mid-polar DB-17HT (J&W Scientific, Folsom, CA, USA) with 60 m column length, 0.25 mm i.d., 0.25 μm film thickness, was used. The oven temperature was programmed from 60 °C (2.5 min held) to 100 °C at a rate of 5 °C/min, from 100 to 225 °C at a rate of 2.5 °C/min and from 225 to 275 °C at a rate of 5 °C, 10 min held at the final temperature was applied. Helium (99.999%) was used as a carrier gas at a constant flow rate of 0.8 mL/min. The split ratio was 1:125, the inlet temperature was set to 260 °C and the transfer line temperature was 280 °C. Mass selective detector operated in electron impact ionization (EI) mode at 70 eV electron energy, the ion source temperature was set to 230 °C and the quadrupole temperature was 150 °C. The mass scan range was 45–1050 *m*/*z*.

#### 3.3.2. Gas Chromatography with Flame-Ionization Detector (GC-FID)

The GC-FID analysis was performed on the same instrument under the same temperature gradient as described above. The system is equipped with a post-column split of the flow, allowing simultaneous analysis on both detectors. Instrument control and data collection were carried out using Mass Hunter Workstation Software (Revision B.06.07, Agilent Technologies).

#### 3.3.3. Identification, Quantitative Analysis and Chemometrics

The identification of the compounds was performed using commercial mass spectral libraries (NIST 14, Wiley 7th Mass spectra register) and retention times (Linear retention indices, LRI). In the cases of a lack of the corresponding reference data, the structures were proposed based on their general fragmentation pattern and/or using the reference literature mass spectra. The quantification of the main compounds was carried out using an internal normalisation method with response factor set equal to unity for all of the sample constituents. Although not being considered as a true quantification, a simple GC-FID percentage allows for a comparison between the studied rose extract samples.

## 4. Conclusions

The liquefied gases extraction of rose flowers yields aroma products, which could be considered as a promising alternative to the traditional rose oil and absolute. The chemical composition of subcritical (1,1,1,2-tetrafluoroethane) extracts from the major oil-bearing rose species, namely, *R. damascena* Mill., *R. gallica* L., *R. centifolia* L. and *R. alba* L., investigated by means of gas chromatography (CG/FID and GC/MS), has revealed that the studied extracts show a similar qualitative content with different quantitative characteristics. The representatives of the phenylethanoids and phenylpropanoids, aliphatic hydrocarbons and terpene compounds (mono-, sesqui- and triterpenoids) are the main constituents. The chemical composition is dominated by *2*-phenylethyl alcohol in the range of 9.0–59.3%. *R. alba* and *R. centifolia* extracts have very similar chemical profiles dominated by the saturated and unsaturated aliphatic hydrocarbons, followed by monoterpenes, phenylethanoids and sesquiterpenes. The *R. damascena* extract is the richest in phenylethanoids with 60.9%, followed by aliphatic hydrocarbons and monoterpenes. The extract of *R. gallica* has an almost equal quantity of aliphatic hydrocarbons and phenylethanoids, the next are mono- and sesquiterpenes. The chemical profile of the subcritical extracts brings them closer to the rose absolute and could be successfully used as its green alternative in cosmetics and aromatherapy.

## Figures and Tables

**Table 1:** Chemical composition of R134a rose extracts, as determined using GC/MS/FID on a DB-17HT column.

No	Compound	LRI_exp_ DB-17HT	Rel.%, as Determined Using GC-FID			
			*R. galica*	*R. damascena*	*R. alba*	*R. centifolia*
1.	α-Pinene	845	0.24	0.42	0.12	0.16
2.	β-Pinene	944	n.d.	0.08	0.01	0.02
3.	β-Myrcene	968	0.17	0.09	0.03	0.02
4.	Limonene	1003	n.d.	n.d.	0.08	0.01
5.	p-Cymene	1026	n.d.	n.d.	0.01	n.d.
6.	Benzaldehyde	1051	n.d.	n.d.	n.d.	n.d.
7.	Linalool	1201	0.07	0.07	0.10	n.d.
8.	Rose oxide	1205	n.d.	0.01	n.d.	n.d.
9.	Benzyl alcohol	1223	0.84	1.48	4.43	0.27
10.	Octanoic acid	1298	0.14	n.d.	0.02	n.d.
11.	2-Phenyl ethyl alcohol	1317	27.22	59.15	14.07	8.99
12.	β-Citronellol	1366	1.72	5.27	2.84	2.85
13.	Nerol	1374	2.24	1.49	6.13	1.26
14.	Phenyl ethyl formate	1381	0.12	0.12	0.08	n.d.
15.	Geraniol	1414	8.46	3.01	14.41	2.87
16.	Neral	1392	n.d.	0.21	0.07	0.10
17.	Geranial	1440	0.34	0.24	0.95	0.09
18.	Phenyl ethyl acetate	1469	n.d.	0.15	0.04	0.11
19.	β-Elemene	1472	n.d.	0.12	0.01	n.d.
20.	Cytronellyl acetate	1475	n.d.	n.d.	0.08	n.d.
21.	Anethole (Benzene,1-methoxy-4(1-propenyl))	1482	n.d.	n.d.	0.25	n.d.
22.	Pentadecane(C15)	1500	0.07	0.06	0.09	n.d.
23.	β-Caryophyllene	1506	0.25	0.17	0.98	0.16
24.	Geranic acid	1522	0.11	0.48	0.26	0.37
25.	β-Copaene	1520	n.d.	n.d.	n.d.	0.26
26.	α-Guaiene	1518	n.d.	0.06	n.d.	n.d.
27.	Geranyl acetate	1540	0.17	0.16	0.03	n.d.
28.	α-Caryophyllene	1552	n.d.	n.d.	n.d.	n.d.
29.	Hydroxy linalool	1568	n.d.	n.d.	0.03	n.d.
30.	Eugenol	1574	0.07	1.26	0.08	n.d.
31.	Germacrene D	1593	0.09	0.11	n.d.	0.74
32.	β-Cubebene	1598	n.d.	n.d.	n.d.	n.d.
33.	α-Muurolene	1607	n.d.	n.d.	n.d.	n.d.
34.	β-Guaiene	1611	n.d.	0.06	n.d.	n.d.
35.	β-Copaene	1616	n.d.	n.d.	n.d.	n.d.
36	β-Cadinene	1646	n.d.	n.d.	n.d.	0.12
37.	Methyl eugenol	1654	n.d.	0.49	n.d.	n.d.
38.	Heptadecane (C17)	1700	1.48	0.46	1.65	0.88
39.	Bulnesol	1703	0.81	0.09	0.25	1.10
40.	Tetradecanal(Myristyl aldehyde)	1706	0.10	n.d.	n.d.	n.d.
41.	Heptadecene (C17:1)	1710	n.d.	n.d.	0.01	n.d.
42.	Benzyl tiglate + Heptadecadiene (C17:2)	1714	0.14	n.d.	0.07	0.27
43.	Octadecane(C18)	1744	0.14	0.06	n.d.	0.07
44.	γ-Eudesmol	1796	n.d.	n.d.	0.02	0.05
45.	τ-Cadinol	1805	n.d.	n.d.	n.d.	n.d.
46.	α-Eudesmol	1819	0.24	n.d.	0.02	0.49
47.	β-Eudesmol	1826	0.36	n.d.	0.14	0.81
48.	α-Cadinol	1833	n.d.	n.d.	0.09	n.d.
49.	Nonadecane + Nonadecene (C19 + C19:1)	1900	17.97	5.09	15.66	12.75
50.	Hexadecanal	1936	0.07	n.d	0.04	n.d
51.	Eicosane(C20)	2000	0.98	0.46	1.50	0.65
52.	Selina-4.7-diol	2046	0.08	n.d	n.d	0.43
53.	Unknown sesquiterpene	2054	0.31	n.d	n.d	0.92
54.	Heneicosane(C21)	2100	9.67	3.09	11.83	8.34
55.	Heneicosene(C21:1)	2105	0.24	0.17	1.31	0.34
56.	Heneicosene(C21:1), isomer	2121	n.d	0.03	0.25	0.07
57.	Docosane(C22)	2200	0.47	0.12	n.d	0.44
58.	Docosene(C22:1)	2211	n.d	n.d	n.d	0.06
59.	Eudesm-4-en-3-one,11-hydroxy	2243	4.67	n.d	n.d	0.11
60.	Tricosane(C23)	2300	0.07	1.27	2.46	8.63
61.	Tricosene(C23:1)	2318	0.30	0.18	1.86	1.65
62.	Tricosene(C23:1), isomer	2334	0.10	0.21	0.33	0.35
63.	1,1,9-Eicosadiene	2348	0.09	0.04	0.12	0.08
64.	Tetracosane (C24)	2400	0.25	0.09	n.d	0.44
65.	Farnesol acetate	2423	0.14	n.d	n.d	n.d
66.	Hexanoic acid,2-ethyl,tetradecyl ester	2449	0.33	0.09	n.d	n.d
67.	Pentacosene(C25:1)	2511	0.22	0.07	0.21	0.17
68.	Pentacosene(C25:1)	2524	0.07	0.05	0.97	0.88
69.	Pentacosene(C25:1)	2532	n.d	n.d	0.32	0.30
70.	Octanoic acid, tetradecyl ester	2548	0.10	0.07	0.04	n.d
71.	Hexacosane(C26)	2600	0.10	0.06	0.05	0.11
72.	Hexacosene(C26:1)	2612	n.d	0.05	0.20	0.13
73.	Octanoic acid, hexadecyl ester	2668	n.d	n.d	n.d	0.22
74.	Heptacosane +Heptacosene (C27 + C27:1)	2700	0.10	0.08	0.21	0.80
75.	Heptacosene(C27:1), isomer	2708	0.42	0.32	0.27	0.33
76.	Heptacosene(C27:1), isomer	2721	n.d	n.d	1.19	1.93
77.	Heptacosanol	2789	n.d	n.d	n.d	0.10
78.	Citronellyl ester (stearate)	2843	0.08	0.18	n.d	0.25
79.	Geranyl ester	2876	n.d	n.d	n.d	0.10
80.	Nonacosene(C29:1)	2911	1.03	0.81	0.49	2.13
81.	Phenyl ethyl ester	2925	0.33	0.52	0.52	0.71
82.	Squalene	2931	0.62	0.08	n.d	0.55
83.	Dodecanoic acid, phenyl methyl ester	2939	n.d	n.d	n.d	0.16
84.	Citronellyl ester (phenyl acetate)	2942	n.d	n.d	n.d	1.28
85.	Neryl ester (phenyl acetate)	2956	n.d	0.06	n.d	1.15
86.	Neryl ester	2961	n.d	n.d	n.d	7.35
87.	Phenyl ethyl ester	2964	n.d	n.d	n.d	1.95
88.	Phenyl ethyl ester (linoleate)	2984	0.08	0.29	n.d	0.10
89.	Citronellyl ester	3016	n.d	n.d	n.d	0.55
90.	Geranyl ester (stearate)	3058	n.d	n.d	n.d	0.87
91.	Neryl ester (stearate)	3076	0.07	0.06	n.d	4.82
92.	Phenyl ethyl ester (stearate)	3112	n.d	n.d	n.d	1.83
93.	Citronellyl ester	3224	n.d	n.d	n.d	0.54
94.	Phenyl ethyl ester	3421	0.13	n.d	n.d	0.45
95.	Neryl ester	3432	n.d	0.06	0.29	0.86
96.	β-Amyrin	3442	0.93	0.45	n.d	0.14
97.	Phenyl ethyl ester	3456	0.31	0.12	n.d	0.35
98.	9,19-Cyclolanost-24-en-3-ol acetate	3462	n.d	n.d	n.d	0.27
99.	Olean-12-en-3-one (Amirenone)	3468	0.27	0.28	0.15	0.65
100.	α-Amyrin + Unindentified triterpene	3476	0.14	0.17	0.09	0.78
101.	Unindentified triterpene	3519	0.79	0.23	n.d	0.25
102.	Lup20(29)-en-3-one	3546	0.20	0.35	n.d	n.d
103.	Lupeol	3616	0.74	1.17	0.15	0.19
Phenylethanoids and phenylpropanoids			27.29	60.90	14.40	8.99
Monoterpenes			13.67	11.89	25.44	25.52
-Monoterpene hydrocarbons			0.41	0.59	0.25	0.21
-Oxygeneted monoterpenes			13.26	11.30	25.19	25.31
Aliphatic hydrocarbons			33.92	12.77	40.98	41.70
Sesquiterpenes			6.95	0.61	2.05	5.19
Triterpenes			3.69	2.73	0.38	2.83
Others			1.15	1.48	4.96	0.38
TOTAL identified			89.81	92.54	89.73	93.68

## Data Availability

The data presented in this study are available in this article.

## Supplementary Materials

The following are available online, Figure S1: Rose species blossom, Figure S2: *R. damascena* raw material, Figure S3: *R. damascena* aroma products.

## Sample Availability

Samples of the compounds are not available from the authors.

## References

[1] Panda H. (2006). Cultivation and Utilization of Aromatic Plants.

[2] Kovacheva N., Zheljazkov V.D., Astatkie T. (2011). Productivity, Oil Content, Composition, and Bioactivity of Oil-Bearing Rose Accessions. HortScience.

[3] Pal P.K. (2013). Evaluation, Genetic Diversity, Recent Development of Distillation Method, Challenges and Opportunities of *Rosa damascena*: A Review. J. Essent. Oil Bear. Plants.

[4] Mileva M., Ilieva Y., Jovtchev G., Gateva S., Zaharieva M.M., Georgieva A., Dimitrova L., Dobreva A., Angelova T., Vilhelmova-Ilieva N. (2021). Rose Flowers—A Delicate Perfume or a Natural Healer?. Biomolecules.

[5] Mahboubi M. (2016). *Rosa damascena* as Holy Ancient Herb with Novel Applications. J. Tradit. Complement. Med..

[6] Ulusoy S., Boşgelmez-Tınaz G., Seçilmiş-Canbay H. (2009). Tocopherol, Carotene, Phenolic Contents and Antibacterial Properties of Rose Essential Oil, Hydrosol and Absolute. Curr. Microbiol..

[7] Wabner D., Beier C. (2012). Aromatherapie: Grundlagen-Wirkprinzipien-Praxis.

[8] Ayati Z., Ramezani M., Amiri M.S., Sahebkar A., Emami S.A., Barreto G.E., Sahebkar A. (2021). Genus Rosa: A Review of Ethnobotany, Phytochemistry and Traditional Aspects According to Islamic Traditional Medicine (ITM). Pharmacological Properties of Plant-Derived Natural Products and Implications for Human Health.

[9] Kovacheva N., Rusanov K., Atanassov I. (2010). Industrial Cultivation of Oil Bearing Rose and Rose Oil Production in Bulgaria During 21st Century, Directions and Challenges. Biotechnol. Biotechnol. Equip..

[10] International Organization for Standardization (2003). ISO Standard 9842:2003 Oil of Rose (Rosa x damascena Miller).

[11] Babu K.G.D., Singh B., Joshi V.P., Singh V. (2002). Essential Oil Composition of Damask Rose (*Rosa damascena* Mill.) Distilled under Different Pressures and Temperatures. Flavour Fragr. J..

[12] Buccellato F. (1980). An Anatomy of Rose. Perfum. Flavorist.

[13] Dobreva A., Getchovska K., Nedeltcheva-Antonova D. (2020). A Comparative Study of Saudi Arabia and Bulgarian Rose Oil Chemical Profile: The Effect of the Technology and Geographic Origin. Flavour Fragr. J..

[14] Dobreva A., Kovacheva N. (2010). Daily Dynamics of Essential Oils of *Rosa damascena* Mill. and *Rosa alba* L.. Agric. Sci. Technol..

[15] Garnero J., Guichard G., Buil P. (1976). L’huile Essentielle et La Concrete de Rose de Turquie. Riv. Ital. Essenze Profumi Piante Off. Aromi Saponi Cosmet. Aerosol.

[16] Koksall N., Aslancan H., Sadighazadi S., Kafkas E. (2015). Chemical Investigation on *Rosa damascena* Mill. Volatiles: Effects of Storage and Drying Conditions. Acta Sci. Pol. Hortorum Cultus.

[17] Kováts E. (1987). Composition of Essential Oils. J. Chromatogr. A.

[18] Nedkov N., Dobreva A., Kovacheva N., Bardarov V., Velcheva A. (2009). Bulgarian Rose Oil of White Oil-Bearing Rose. Bulg. J. Agric. Sci..

[19] Ohloff G., Demole E. (1987). Importance of the Odoriferous Principle of Bulgarian Rose Oil in Flavour and Fragrance Chemistry. J. Chromatogr. A.

[20] Pellati F., Orlandini G., van Leeuwen K.A., Anesin G., Bertelli D., Paolini M., Benvenuti S., Camin F. (2013). Gas Chromatography Combined with Mass Spectrometry, Flame Ionization Detection and Elemental Analyzer/Isotope Ratio Mass Spectrometry for Characterizing and Detecting the Authenticity of Commercial Essential Oils of *Rosa damascena* Mill.: GC/MS, GC/FID and GC/C/IRMS Analysis of *Rosa damascena* Essential Oil. Rapid Commun. Mass Spectrom..

[21] Xiao Z., Luo J., Niu Y., Wu M. (2018). Characterization of Key Aroma Compounds from Different Rose Essential Oils Using Gas Chromatography-Mass Spectrometry, Gas Chromatography–Olfactometry and Partial Least Squares Regression. Nat. Prod. Res..

[22] Cebi N., Arici M., Sagdic O. (2021). The Famous Turkish Rose Essential Oil: Characterization and Authenticity Monitoring by FTIR, Raman and GC–MS Techniques Combined with Chemometrics. Food Chem..

[23] Aydinli M., Tutaș M. (2003). Production of Rose Absolute from Rose Concrete. Flavour Fragr. J..

[24] Nedeltcheva-Antonova D., Stoicheva P., Antonov L. (2017). Chemical Profiling of Bulgarian Rose Absolute (*Rosa damascena*Mill.) Using Gas Chromatography–Mass Spectrometry and Trimethylsilyl Derivatives. Ind. Crops Prod..

[25] Ohashi T., Miyazawa Y., Ishizaki S., Kurobayashi Y., Saito T. (2019). Identification of Odor-Active Trace Compounds in Blooming Flower of Damask Rose (*Rosa damascena*). J. Agric. Food Chem..

[26] Aycı F., Aydınlı M., Bozdemir Ö.A., Tutaş M. (2005). Gas Chromatographic Investigation of Rose Concrete, Absolute and Solid Residue. Flavour Fragr. J..

[27] Krupčík J., Gorovenko R., Špánik I., Sandra P., Armstrong D.W. (2015). Enantioselective Comprehensive Two-Dimensional Gas Chromatography. A Route to Elucidate the Authenticity and Origin of *Rosa damascena* Miller Essential Oils: Gas Chromatography. J. Sep. Sci..

[28] Kurkcuoglu M., Baser K.H.C. (2003). Studies on Turkish Rose Concrete, Absolute, and Hydrosol. Chem. Nat. Compd..

[29] Özkan G., Sagdiç O., Baydar N.G., Baydar H. (2004). Antioxidant and Antibacterial Activities of *Rosa damascena* Flower Extracts. Food Sci. Technol. Int..

[30] Chemat F., Rombaut N., Fabiano-Tixier A.-S., Pierson J.T., Bily A., Chemat F., Strube J. (2015). Green Extraction: From Concepts to Research, Education, and Economical Opportunities. Green Extraction of Natural Products.

[31] Gochev V., Girova T., Stoilova I., Atanasova T., Nenov N., Stanchev V., Stoyanova A. (2012). Low Temperature Extraction of Essential Oil Bearing Plants by Liquefied Gases. 7. Seeds from Cardamom (Elettaria Cardamomum (L.) Maton). J. Biosci. Biotechnol..

[32] Khambay B.P.S. Extraction and Isolation of Artemisin with HFC-134a. Proceedings of the Artemisinin Forum 2008.

[33] Mihov R., Nikovska K., Nenov N., Slavchev A. (2012). Evaluation of Mayonnaise-like Food Emulsions with Extracts of Herbs and Spices. Emir. J. Food Agric..

[34] Nenov N., Gochev V., Girova T., Stoilova I., Atanasova T., Stanchev V., Stoyanova A. (2011). Low Temperature Extraction of Essential Oil Bearing Plants by Liquefied Gases. 6. Barks from Cinnamon (*Cinnamomum Zeylanicum* Nees). J. Essent. Oil Bear. Plants.

[35] Stoyanova A., Nenov N., Slavchev A., Jirovetz L., Buchbauer G., Lien H., Schmidt E., Geissler M. (2006). C2H2F4-Oleoresins of Black Pepper(Piper NigrumL.)and Ginger (Zingiber Officinale(L.)Rosc.) from Vietnam: Antimicrobial Testings, Gas Chromatographic Analysis and Olfactoric Evaluation. Electron. J. Environ. Agric. Food Chem..

[36] (2009). Directive 2009/32/EC of the European Parliament and of the Council of 23 April 2009 on the Approximation of the Laws of the Member States on Extraction Solvents Used in the Production of Foodstuffs and Food Ingredients. Off. J. Eur. Union.

[37] (2018). Solkane^®^134a Thermodynamics.

[38] (2018). SOLKANE^®^ 227 Pharma SOLKANE^®^ 134a Pharma.

[39] Haghighi A., Khajenoori M., Nakajima H. (2013). Subcritical Water Extraction. Mass Transfer-Advances in Sustainable Energy and Environment Oriented Numerical Modeling.

[40] Özel M.Z., Göğüş F., Lewis A.C. (2006). Comparison of Direct Thermal Desorption with Water Distillation and Superheated Water Extraction for the Analysis of Volatile Components of *Rosa damascena* Mill. Using GCxGC-TOF/MS. Anal. Chim. Acta.

[41] Manouchehri R., Saharkhiz M.J., Karami A., Niakousari M. (2018). Extraction of Essential Oils from Damask Rose Using Green and Conventional Techniques: Microwave and Ohmic Assisted Hydrodistillation versus Hydrodistillation. Sustain. Chem. Pharm..

[42] Reverchon E., Delta Porta G. (1996). Rose Concrete Fractionation by Supercritical CO2. J. Supercrit. Fluids.

[43] Boelens M.H., Boelens H. (1997). Differences in Chemical and Sensory Properties of Orange Flower and Rose Oils Obtained from Hydrodistillation and from Supercritical C02 Extraction. Perfum. Flavorist.

[44] Reverchon E., Della Porta G., Gorgoglione D. (1997). Supercritical CO2 Extraction of Volatile Oil from Rose Concrete. Flavour Fragr. J..

[45] Ghoreishi S.M., Hedayati A., Mousavi S.O. (2016). Quercetin Extraction from *Rosa damascena* Mill via Supercritical CO2: Neural Network and Adaptive Neuro Fuzzy Interface System Modeling and Response Surface Optimization. J. Supercrit. Fluids.

[46] Wilde P.F., McClory P.J. (1994). New Solvents for Extraction. Perfum. Flavorist.

[47] Baser K.H.C., Kurkcuoglu M., Özek T. (2003). Turkish Rose Oil Research: Recent Results. Perfum. Flavorist.

[48] Antonova D.V., Medarska Y.N., Stoyanova A.S., Nenov N.S., Slavov A.M., Antonov L.M. (2020). Chemical Profile and Sensory Evaluation of Bulgarian Rose (*Rosa damascena* Mill.) Aroma Products, Isolated by Different Techniques. J. Essent. Oil Res..

[49] Nenov N., Atanasova T., Gochev V., Merdzhanov P., Girova T., Djurkov T., Stoyanova A. (2016). New Product from Bulgarian Rose. Int. Sci. Pract. Conf. World Sci..

[50] Roth M. (1996). Thermodynamic Prospects of Alternative Refrigerants as Solvents for Supercritical Fluid Extraction. Anal. Chem..

[51] Joichi A., Yomogida K., Awano K., Ueda Y. (2005). Volatile Components of Tea-Scented Modern Roses and Ancient Chinese Roses. Flavour Fragr. J..

[52] Hirata H., Ohnishi T., Watanabe N. (2016). Biosynthesis of Floral Scent 2-Phenylethanol in Rose Flowers. Biosci. Biotechnol. Biochem..

[53] Rusanov K., Kovacheva N., Rusanova M., Atanassov I. (2012). Reducing Methyl Eugenol Content in *Rosa damascena* Mill Rose Oil by Changing the Traditional Rose Flower Harvesting Practices. Eur. Food Res. Technol..

[54] Tan K.H., Nishida R. (2012). Methyl Eugenol: Its Occurrence, Distribution, and Role in Nature, Especially in Relation to Insect Behavior and Pollination. J. Insect Sci..

[55] Dobreva A., Velcheva A., Bardarov V., Bardarov K. (2013). Chemical Composition of Different Genotypes Oil - Bearing Roses. Bulg. J. Agric. Sci..

[56] Rusanov K., Kovacheva N., Atanassov I. (2011). Comparative GC/MS Analysis of Rose Flower and Distilled Oil Volatiles of The Oil Bearing Rose *Rosa damascena*. Biotechnol. Biotechnol. Equip..

[57] Ghosh S. (2020). Triterpenoids: Structural diversity, biosynthetic pathway, and bioactivity. Studies in Natural Products Chemistry.

[58] Riffault-Valois L., Destandau E., Pasquier L., André P., Elfakir C. (2016). Complementary Analytical Methods for the Phytochemical Investigation of ‘Jardin de Granville’, a Rose Dedicated to Cosmetics. Comptes Rendus Chim..

[59] Da Porto C., Decorti D., Natolino A. (2015). Application of a Supercritical CO _2_ Extraction Procedure to Recover Volatile Compounds and Polyphenols from *Rosa damascena*. Sep. Sci. Technol..

[60] Staikov V., Decheva R., Balinova-Tsvetkova A. (1975). Studies on the Composition of Rose Oil Obtained from the Flowers in Different Stages of Their Development. Riv. Ital..

[61] Seify Z., Yadegari M., Pirbalouti A. (2018). Essential Oil Composition of *Rosa damascena* Mill. Produced With Different Storage Temperatures and Durations. Korean J. Hortic. Sci. Technol..

[62] Barao T., de Castro C.A.N., Mardolcar U.V., Okambawa R., St-Arnaud J.M. (1995). Dielectric Constant, Dielectric Virial Coefficients, and Dipole Moments of 1,1,1,2-Tetrafluoroethane. J. Chem. Eng. Data.

