# Supplementary material for: Subcritical Extracts from Major Species of Oil-Bearing Roses—A Comparative Chemical Profiling
Source: Molecules. 2021 Aug 18;26(16):4991. doi: 10.3390/molecules26164991 (PMC8398789; doi:10.3390/molecules26164991)

**Subcritical Extracts from Major Species of Oil-Bearing Roses - A Comparative Chemical Profiling**

A. Dobreva[1], D. Nedeltcheva-Antonova[2*], N. Nenov[3], K. Getchovska[2] and L. Antonov[4]

[1] Institute of Roses and Aromatic Plants, Agricultural Academy, 6100 Kazanlak, Bulgaria
[2] Institute of Organic Chemistry with Centre of Phytochemistry, Bulgarian Academy of Sciences, 1113 Sofia, Bulgaria
[3] University of Food Technologies, Department of Heat Engineering, Technical Faculty, 4002 Plovdiv, Bulgaria
[4] Institute of Electronics, Bulgarian Academy of Sciences, 1784 Sofia, Bulgaria
*Corresponding author, E-mail: Daniela.Antonova@orgchm.bas.bg

Figure S1. Rose species blossom.

a) *R.alba*

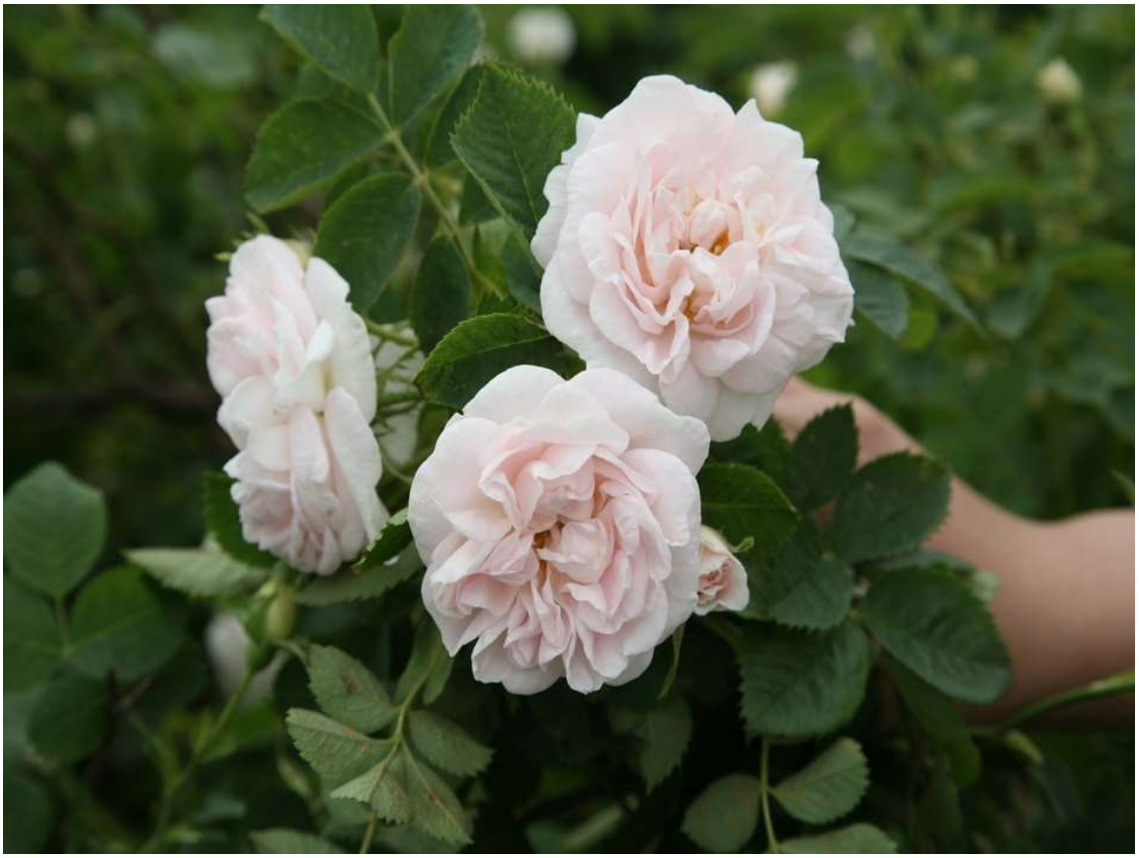

b) *R.centifolia*

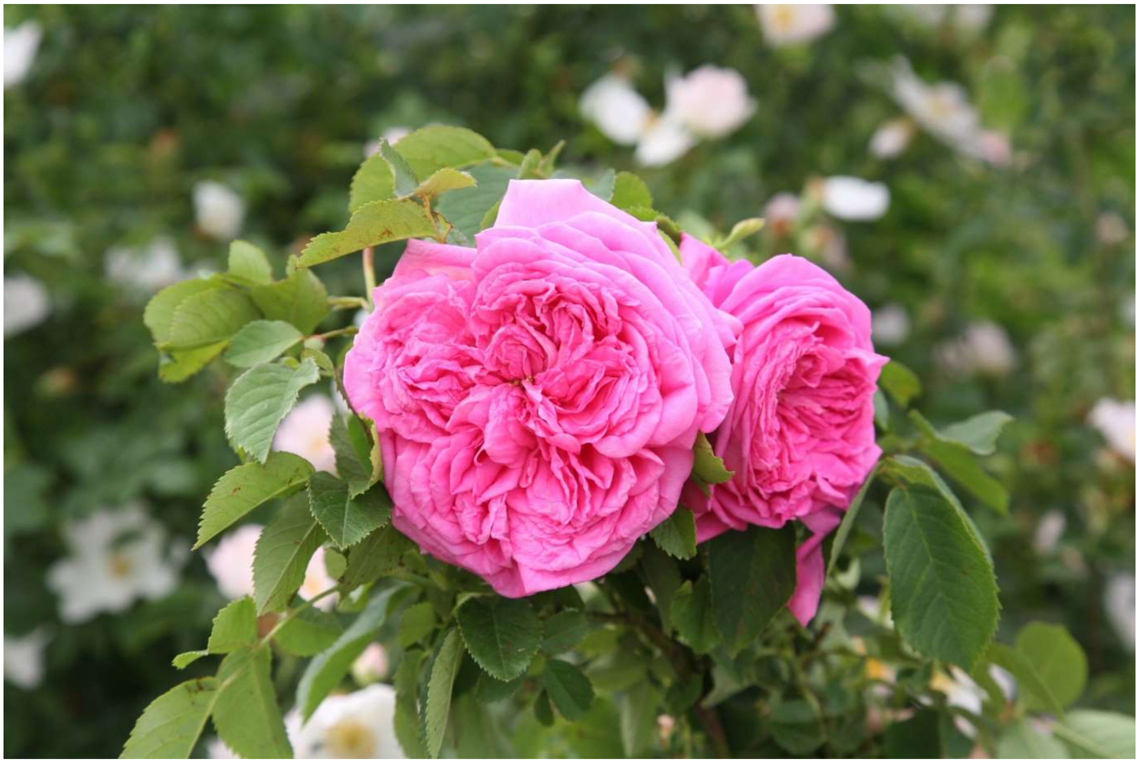

c) *R:damascena*

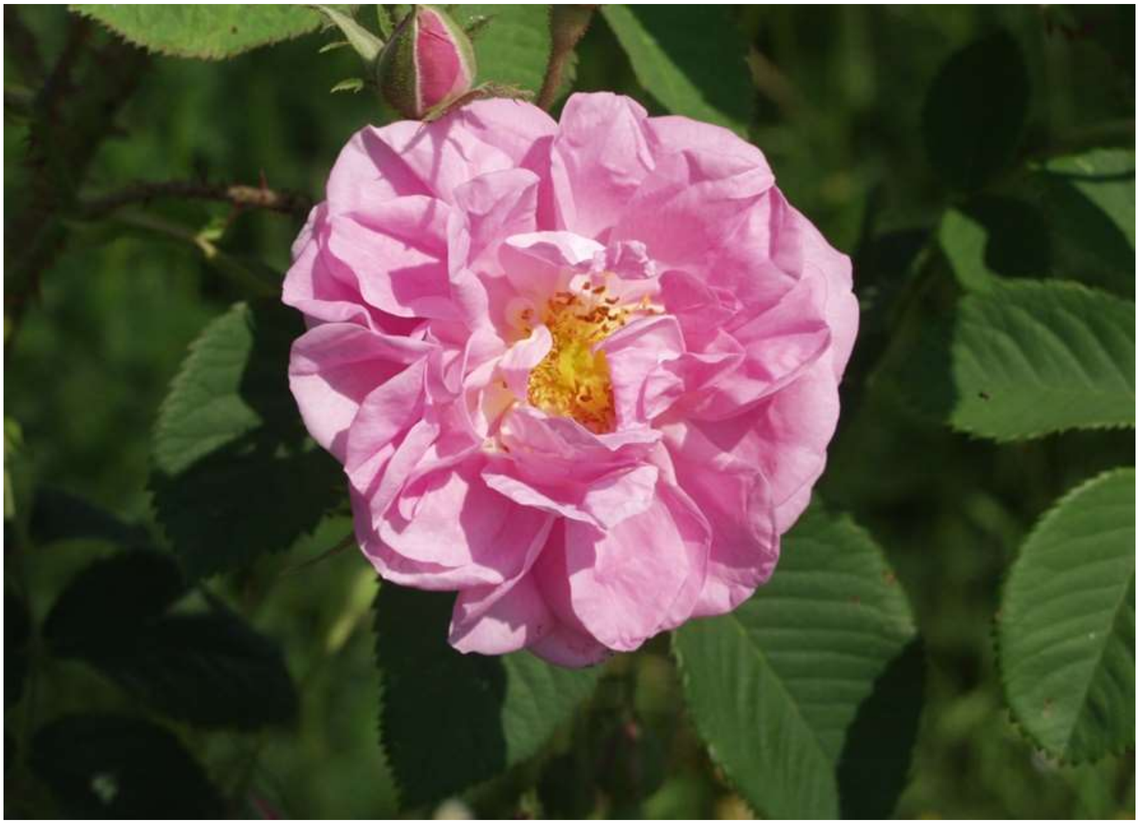

d) *R.gallica*

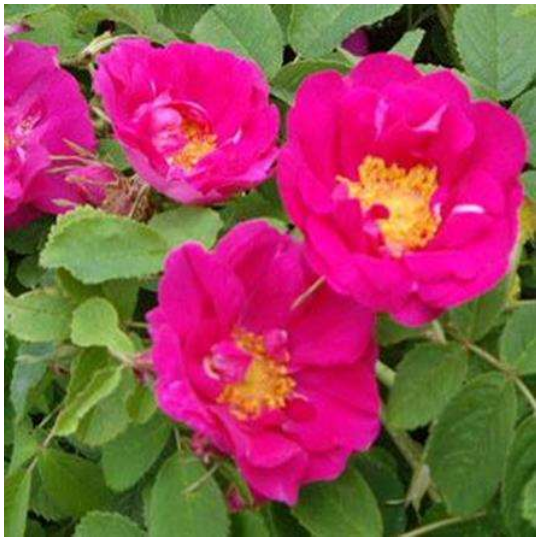

Figure S2. *R.damascena* raw material.

a) *R.damascena* fresh flowers

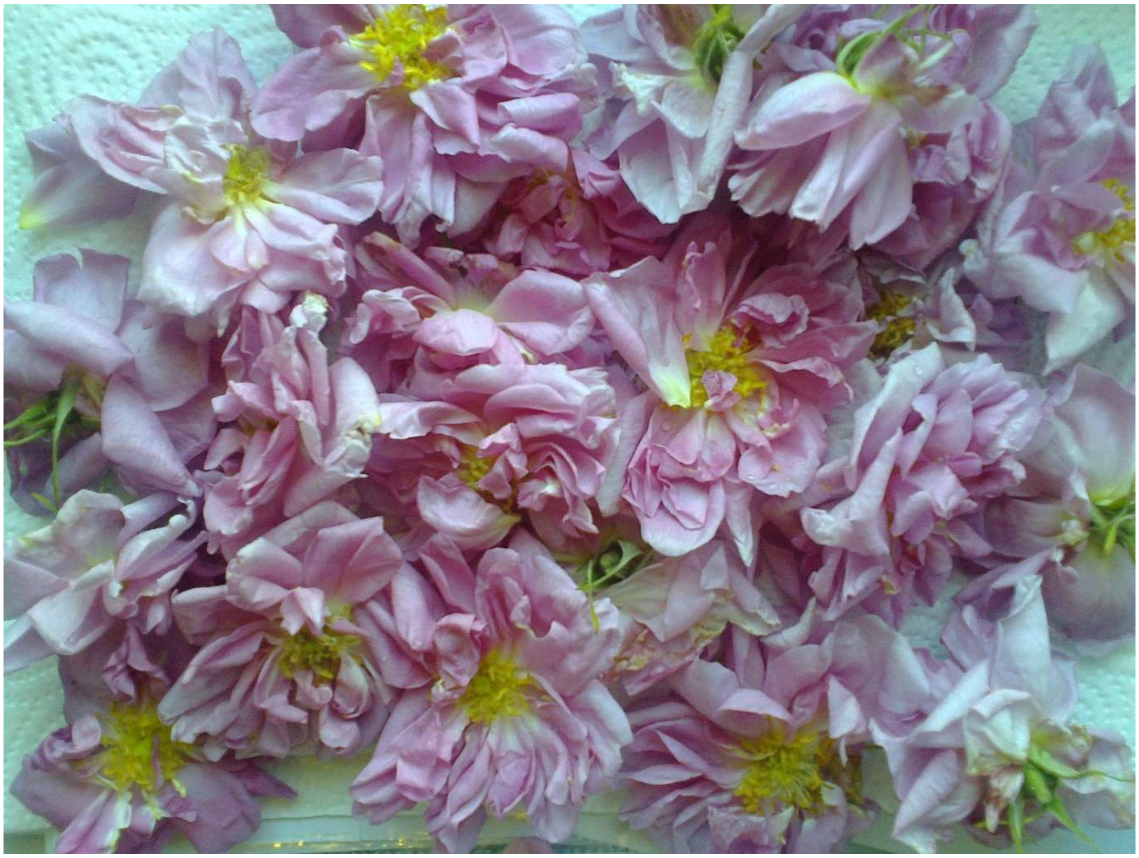

b) *R.damascena* exhausted flowers

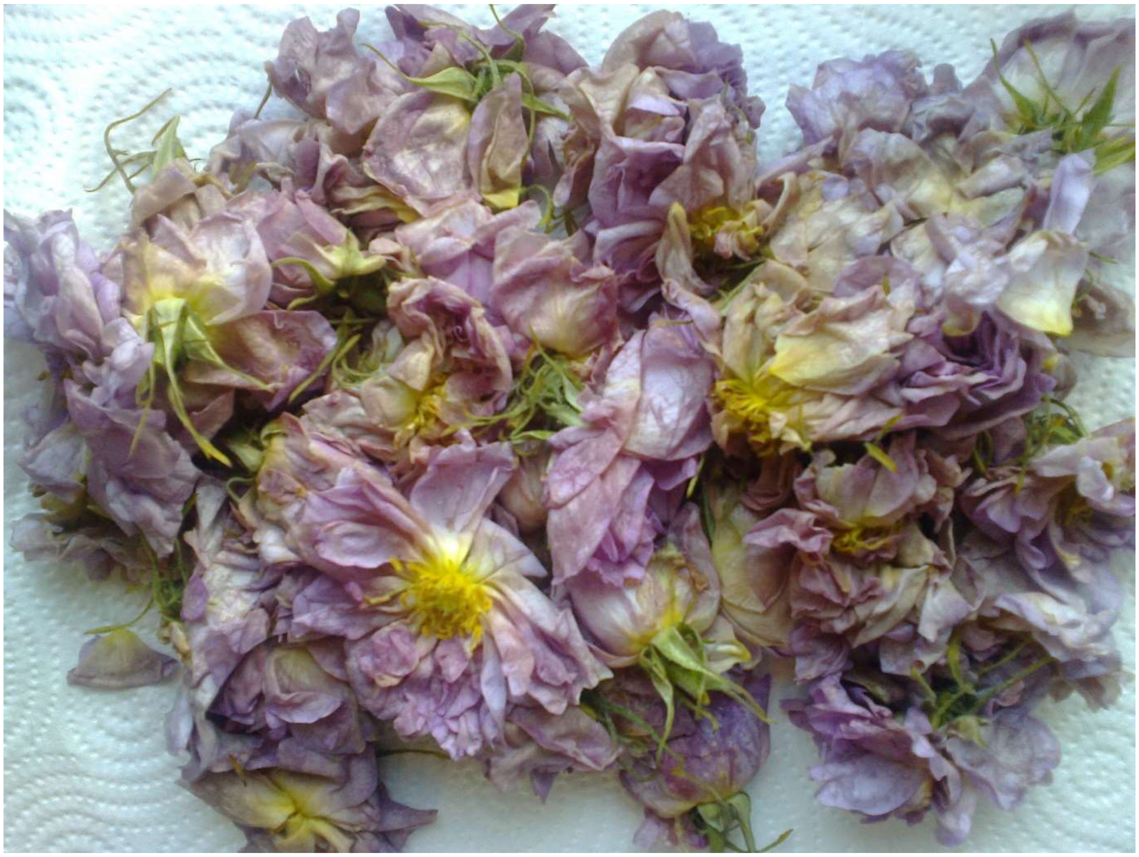

Fig.S3 *R.damascena* aroma products (from left to right – rose absolute, derived by traditional method, essential oil and subcritical extract)

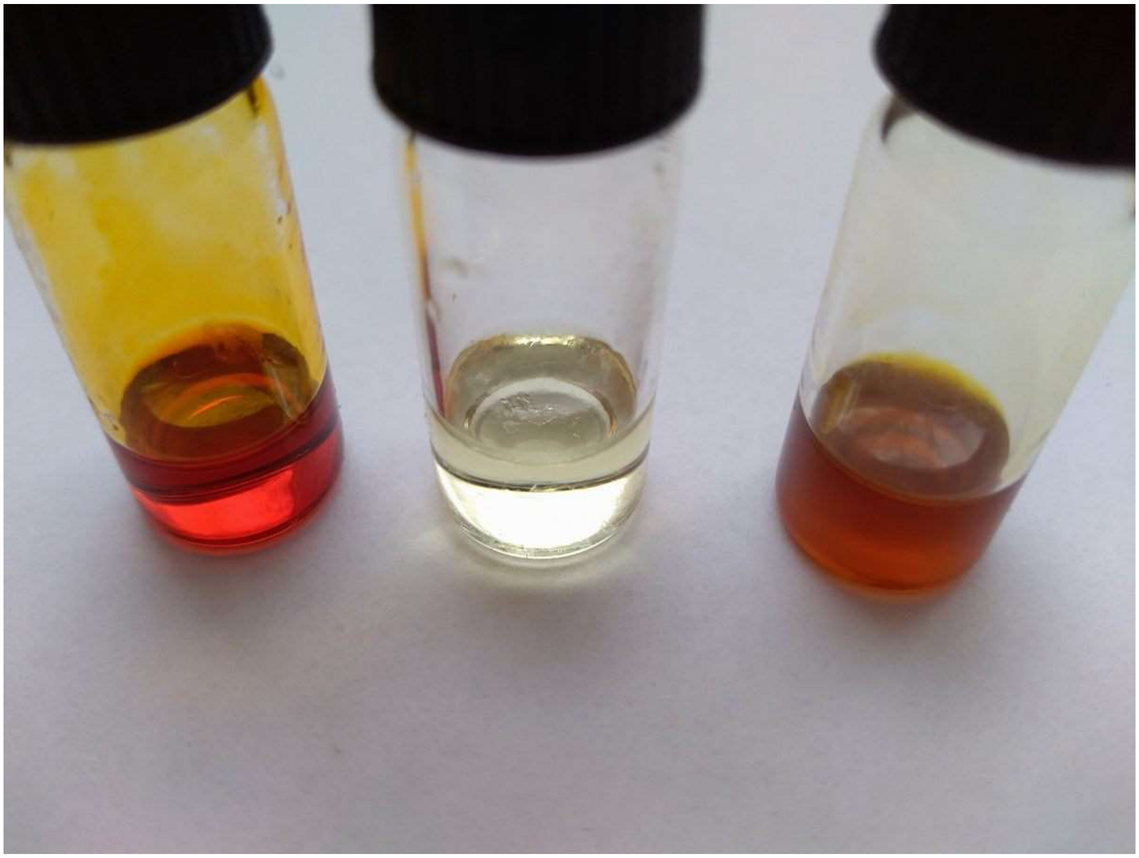

Supplement: Supplementary file 1 [file molecules-26-04991-s001.zip › molecules-1323502-supplementary.pdf]
